# Alliance for Scientific Autism Intervention: System Components and Outcome Data from High-Quality Service Delivery Organizations

**DOI:** 10.1007/s40617-023-00898-7

**Published:** 2023-12-29

**Authors:** Dawn Buffington Townsend, Kevin J. Brothers, Gregory S. MacDuff, Amanda Freeman, Christine Fry, Eric Rozenblat, Donna DeFeo, Anna Budzinska, Iwona Ruta-Sominka, Binyamin Birkan, Laura J. Hall, Patricia J. Krantz, Lynn E. McClannahan

**Affiliations:** Alliance for Scientific Autism Intervention, 381 Madison Avenue, New Milford, NJ 07646 USA

**Keywords:** Autism, Organizational systems, Evidence-based practice, Quality autism education program, Client outcomes

## Abstract

Promoting excellence in autism intervention is arguably more urgent than ever for the field of applied behavior analysis. To fulfill this objective, autism agencies must operate from validated program systems and do so with fidelity. Program components include, but are not limited to, staff training and evaluation of clinical skills, functional personnel roles designed to promote positive outcomes for those served, and professional staff-communication-skill repertoires. Data on client outcomes must be tied to implementation of core program variables; and, contingencies between the data on client outcomes and staff performance must exist. Furthermore, these contingencies must be yoked across members of the organization to ensure a sustainable and effective program model. Finally, data on consumer satisfaction must be collected and used to evaluate program components and agency practices. Members of the Alliance for Scientific Autism Intervention have implemented key program-wide systems based upon the work of McClannahan and Krantz *Journal of Applied Behavior Analysis, 26*, 589–596 (1993) for decades and across various agency cultures. Data collected by six independent educational agencies on client outcomes, program implementation, and consumer feedback for a 10-year time span demonstrate the sustainability of the model and support the importance of key organizational systems and the relationship between implementation of the model and high-quality outcomes for individuals with autism.

At this point in time, there is no question about the importance of providing effective science-based intervention to individuals with autism. There is a wealth of behavior-analytic research that documents how specific intervention practices are associated with socially significant gains in the skill repertoires of individuals with autism (Hume et al., 2021; Slocum et al., 2014). What remains less clear, however, are the variables associated with the development of a high-quality autism-intervention program. Silbaugh and El Fattal (2022) make a compelling argument for the need to define and describe the components of service delivery organizations that consistently produce the desired outcomes for the consumers of the organization, and thereby can be considered high quality. As noted by Silbaugh and El Fattal, it is imperative for the field to move beyond program descriptions (e.g., Handleman & Harris, 2005; McGee et al., 2020) to demonstrations of program effectiveness that include client progress towards functional goals, consumer satisfaction, and overall improvement in quality of life (Dixon, 2014). They argue that it is necessary to define, at an organizational level, quality-dependent key performance indicators (QD-KPIs), strategic plans allowing achievement of these QD-KPIs, organizational systems, and contingencies (both within and external to the organization) that are necessary to ensure high-quality applied behavior analysis (ABA) services. Of equal importance, is the collection of data on the implementation and sustainability of key service delivery components, such as an effective training and evaluation/supervision model, collection of social validity data, and internal accountability and communication systems as noted by both the Council of Autism Service Providers (CASP, 2020) and the Behavioral Health Center of Excellence (BHCOE, 2021) as important quality assurance measures.

To be able to promote the effectiveness of programs based on behavior analytic principles, organizations must define the key variables associated with producing positive outcomes (Baer et al., 1987; McClannahan & Krantz, 1993). Outcomes should be measured against predefined quality standards. Data on adherence to professional standards, program-wide client outcomes, and consumer satisfaction can be used to ascertain effectiveness of a wide variety of systems used by autism agencies (BHCOE, 2021). As noted in both implementation science and organizational behavior management frameworks, this could include (at a minimum) evaluating leadership, the systems of communication, teamwork, data-based decision making, training, and feedback within an autism agency (Odom et al., 2020). A true test of a high-quality service delivery organization is that the desired outcomes are sustained over time.

High-quality service delivery systems must not only have clearly identified and evaluated program components, but these components must be interrelated and operate under individual, group, and yoked contingencies (Glenn & Malott, 2004). It is important to note that these contingencies (such as increased client referrals, tuition income and employee retention) must be tied to program outcomes, including the skill acquisition, progress, and satisfaction of autistic individuals and their families resulting from the aggregate efforts of a group of individuals in the organization (e.g., instructional staff member, trainer, and program director). Furthermore, the implementation of the same organizational components and contingencies operating in multiple programs with similar positive outcomes would provide further evidence of service delivery program effectiveness.

Silbaugh and El Fattal (2022) propose a “call to action” encouraging ABA service providers to implement six steps that benefit an organization in moving towards the provision of high-quality services. Although there is much work to be done, as noted by these authors, they suggest organizations begin working on making practical changes. These six steps include: (1) strategic planning; (2) definition of QD-KPIs; (3) monitoring of progress; (4) inclusion of both professional and consumer standards in the QD-KPIs; (5) total quality management system implementation; and (6) regular review—and publishing—of results (see Silbaugh & El Fattal, 2022, for a full discussion).

For more than 40 years Drs. Krantz and McClannahan not only forged a path of well-articulated organizational components and group contingencies that improved the lives of those they served directly, they also collaborated with behavior analysts around the globe to do the same in new organizations through their dissemination efforts (Krantz & McClannahan, 1999; McClannahan & Krantz, 2005; Williams & Williams, 2010). More recently, they worked to establish the Alliance for Scientific Autism Intervention (ASAI). Members of this organization, all having been mentored by Drs. McClannahan and Krantz, embrace the establishment of the service delivery systems they developed and are committed to being high-quality service-delivery agencies that are aligned with the six steps identified by Silbaugh and El Fattal (2022).

This article describes the organizational systems Drs. McClannahan and Krantz established based on the principles of ABA and their implementation across agencies that have been providing educational services in schools to individuals with autism and their families from as early as 1975 (described more fully in McClannahan & Krantz, 1993). Data are presented across six different ASAI member agencies. Although program outcomes (e.g., Cohen et al., 2006; Eldevik et al., 2006; Fenske et al., 1985; Howard et al., 2005; Smith et al., 2000), and a detailed description of the organizational systems (McClannahan & Krantz, 1993) have been published, this is the first summary of process and outcome data across various agencies, each operating as an independent organization. Our purpose in sharing these data with a wider audience is to demonstrate that it is possible to consistently implement critical program components, ensure consistency in service delivery, and develop organizational cultures and practices that are linked to positive client outcome measures and consumer satisfaction.

## Alliance for Scientific Autism Intervention (ASAI)

ASAI is dedicated to the preservation and advancement of effective science-based intervention for individuals with autism (see www.ASAI.science). All members of ASAI implement and contribute to the intervention model initially developed by McClannahan and Krantz (1993), collect annual process and outcome data, and present their outcome data annually to all members. ASAI has a member-elected Board of Trustees, an executive director, and both full (voting) and affiliate (nonvoting) members. Agencies join ASAI as affiliate members, complete a mentorship process, and obtain full member status after they meet the criteria defined in the ASAI Standards of Excellence as seen Table 1. All ASAI member programs agree to share their annual data with each other and ensure compliance with ethical practices through staff training activities, obtaining informed parental consent, the invitation of an external evaluator to assess compliance with current ethical practices in the field, an active Human Rights Committee, and accountability in consumer and collegial reporting practices.

**Table 1:** ASAI program requirements as defined in the ASAI standards of excellence for becoming and remaining full members

Program component	Minimum criterion each year
Program Director	Active program Director trained in behavior analysis
Instructional Staff	1:2 Instructional staff to client ratio (for the majority of the day)
Training Staff	1:8 Full time Trainer to Instructional staff ratio
Didactic Staff Training	25 hr in behavior analytic content delivered throughout the year
Staff Evaluation	90% of instructional staff evaluated
Home Programming	22 visits per family
Client Performance Data	80% on all dimensions of external notebook review
Program-wide engagement data	80% on-task data across four program-wide observations
Consumer Evaluation	Satisfactory ratings (6) across each area by 80% of each consumer group

Six ASAI member agencies participated in the implementation of the program model and collection of data included in this article (see Table 2). There were five full members and one affiliate member. Four full members were located in the United States and one was located in Poland. The affiliate member was located in Turkey. All agencies operated an 11-month education program, providing educational services to autistic clients from ages 3–21 in accordance with requirements established by the state Department of Education or similar credentialing agency abroad. The majority of clients in all of the programs were placed by either local school districts or guardians because the individuals did not make progress in their then-current placement and/or the extent of the behavioral deficits and excesses prevented them from receiving an appropriate education in a public-school setting. Most of the clients joined the programs between 3 and 8 years of age. Those clients who acquired repertoires enabling them to return to their sending school districts graduated from the agencies. Those who continued to need extensive support and individualized programming remained in the agency education programs (with the exception of Organization E) until the age of 21. All of the agencies provided 30 hr of ABA-based intervention, 5 days a week, as well as guardian/family support services. The agencies in the United States were funded by local or state departments of education and agency fund-raising efforts. The agencies abroad were funded through private pay, fund-raising efforts and minimal governmental support. All five full member agencies collected data across a 10-year period, from July 1, 2011 to June 30, 2021. The affiliate member collected data for 2 years, since its inception in 2019.

**Table 2:** Member agency information from 2011 to 2021

Agency name	Location	Number of years in operation in 2021	Mean number (and range) of clients per year	Age range of clients (years)
Full Member A	USA	51	31 (29–34)	3–21
Full Member B	USA	28	26 (24–29)	3–21
Full Member C	USA	25	28 (25–30)	3–21
Full Member D	USA	22	30 (28–30)	3–21
Full Member E	Poland	15	20 (14–22)	3–9
Affiliate Member	Turkey	2	11 (8–13)	3–21

## Key Performance Indicators and Strategies Likely to Result in Attainment

Silbaugh and El Fattal (2022) identify the first two steps in their call to action as the need to establish strategic plans and QD-KPIs necessary to accomplish an organization’s strategic goals. The ASAI agencies’ QD-KPIs, listed in Table 3, include measures of client progress towards defined goals and the satisfaction of consumers involved with the agency. These QD-KPIs are used to assess attainment of delivering high-quality services that are based on both professional and consumer standards. Of equal importance is the need to use standardized, well-defined, and consistent data collection methods across agencies to allow for a comparison of outcomes relative to system components (BHCOE, 2021). Regular, annual collection and review of standard, but individual, client-based data across multiple agencies enables analysis of the extent to which high-quality outcomes are attained over agencies and across time, regardless of shifts in cultural, social, and other factors (including the COVID-19 pandemic). In addition, these data indicate needed areas of improvement and future strategic development. The collection and organization of these data align with Silbaugh and El Fattal’s Step 3, recommending the establishment of a dashboard that allows leadership to monitor progress over time, not only in an individual agency but across multiple agencies, with respect to attainment of QD-KPIs.

**Table 3:** Definition of agency systems along with related goals and QD-KPIs

Goal	QD-KPI	Target	System
Positive client outcomes in the education setting	Percentage of skill acquisition and behavior decrease programs scored as effective	80%^a^	External evaluation
Establish beneficial behavioral repertoires at home	Percentage of home programs scored as effective	80%	External evaluation
Develop defined clinical and professional staff repertoires	Percentage of staff passing the performance evaluation	90%	Annual staff evaluation
Provide regular hands-on staff training and support	Number of instructional staff assigned to a trainer	8	Organizational structure
Ensure individualized & sufficient teaching opportunities	Instructional staff to client ratio	1:2	Organizational structure
Guardian satisfaction	Percentage of guardians satisfied	80%	Guardian survey
Staff satisfaction with program administration	Percentage of staff satisfied	80%	Staff survey
Staff satisfaction with colleague performance	Percentage of staff satisfied	80%	Colleague survey
Placement agency satisfaction	Percentage of agencies satisfied	80%	Placement team survey
Governing board satisfaction	Percentage satisfied	80%	Governing board survey

^a^ Percentage of skill acquisition and behavior decrease programs scored as effective

### Staff Training and Performance Evaluation

Skilled professionals are one of the most important elements of any educational program. Instructional staff must demonstrate proficiency in key areas that are defined and measured, including clinical, professional, data collection, and data analysis skills (Ellis & Glenn, 1995; McClannahan & Krantz, 1981). The ASAI agencies adhere to a consistent model of training and evaluation to promote successful staff development and assessment of performance relative to standards set by the agency, a system that aligns with Step 4 of Silbaugh and El Fattal’s (2022) call to action. Each ASAI agency uses a similar protocol to train and evaluate staff members. A minimum of 25 hr of didactic training in behavior analysis is provided outside the educational context through workshops delivered at hire and in an ongoing manner throughout each academic year. More important, hands-on training within educational programs is a daily occurrence in each agency. Behavioral skills training (Parsons et al., 2012; Sherman et al., 2021) is expected to occur regularly with a trainer modeling skills, providing practice opportunities, and delivering feedback and ongoing practice until criterion is achieved. This high level of daily, in-classroom training requires trainers to be highly skillful themselves, regularly available, and accountable to the instructional staff and clients with respect to producing skill acquisition (McClannahan & Krantz, 1993; Reid et al., 2017).

All ASAI agencies employ instructional staff who are responsible for providing hands-on teaching, collecting and analyzing data, preparing curriculum, and advancing programming for the clients. All instructional staff members have, at minimum, a bachelor’s level degree in education, psychology, or a related field. Each instructional staff member is assigned to a particular client to ensure regular data collection and program oversight for a 1-year period, but works with all of the clients in a classroom throughout a day and is responsible for ensuring client progress for all assigned teaching tasks.

In each ASAI agency, hands-on-training is provided by highly skilled trainers who meet defined performance criteria. One trainer is assigned to each classroom to provide training to instructional staff in that classroom and oversight for client progress for those clients in that classroom. Trainers are staff who hold, at minimum, a bachelor’s degree, have passed at least two staff evaluations, and have demonstrated advanced competence in clinical, professional, and behavior analytic repertoires measured on the staff evaluation protocol. A trainer’s primary responsibilities include providing hands-on training to instructional staff, assessing instructional staff performance through direct observation, reviewing client progress, and meeting with instructional staff members to discuss necessary programming changes. Trainers spend at least 80% of their 40-hr (at minimum) work week completing these responsibilities.

The availability of sufficient training staff is necessary to develop skillful instructional staff members who can pass an evaluation and produce client progress towards goals and objectives, two critical QD-KPIs. As such, each ASAI agency ensures a high in-class trainer to instructional staff ratio and provides each trainer and instructional staff member with the standardized training and assessment tool at the commencement of their employment. This tool is referenced repeatedly throughout training sessions. As can be seen in Table 4, a trainer was responsible for no more than seven staff members in any given year in each agency, ensuring a high trainer to instructional staff (and client) ratio.

**Table 4:** Staff details across agencies from 2011 to 2021

Organization	Number of instructional staff per year		Number of trainers per year		Number of instructional staff assigned to one trainer per year	
	Mean	Range	Mean	Range	Mean	Range
Full Member A	31	26–40	7	4–8	5	4–7
Full Member B	15	11–19	5	4–6	3	2–3
Full Member C	27	25–29	5	3–7	6	4–8
Full Member D	28	27–35	6	5–6	5	4–6
Full Member E	17	13–20	4	3–7	5	4–6
Affiliate Member	12	12–13	4	3–4	4	3–4

The professional staff training and evaluation protocol measures both client performance and instructional staff performance. All ASAI agencies use a professional evaluation process that includes a full 6-hr day of observation of an instructional staff member using the standardized evaluation tool. Direct observation and data collection throughout the 6-hr evaluation assess (1) client engagement; (2) the number of opportunities to respond that are provided by the instructional staff member; (3) the number of behavior-specific praise statements that are provided by the instructional staff member; and (4) the number of incidental teaching episodes and script and script-fading instructional programs used to develop language. Several other areas are evaluated using a 7-point Likert scale ranging from *completely satisfied* (7) to *completely dissatisfied* (1), as seen in Table 5. The areas rated include (1) professionalism; (2) teaching new skills using behavioral strategies and evidence-based practice; (3) increasing social competence; (4) decreasing challenging behavior and teaching functional replacement behavior; (5) developing positive relationships; (6) arranging the environment to promote learning; (7) programming for generalization; (8) discussing intervention technology; and (9) ensuring individualized and effective programming through a complete review of every individualized program and associated data summary in the client’s notebook for which the instructional staff member is responsible.

**Table 5:** Rating scale used for staff evaluation protocol

Rating	Label	Definition	Performance level
7	Completely satisfied	Staff member displays requisite skill above criterion	Above criterion
6	Satisfied	Staff member displays requisite skill at criterion	Criterion
5	Slightly satisfied	Staff member displays some components of skill or displays skill in some situations	Below criterion
4	Neither satisfied or dissatisfied	Staff member acknowledges importance of skill and/or is attempting to display it	Below criterion
3	Slightly dissatisfied	Staff member does not display this skill	Below criterion
2	Dissatisfied	Staff member displays responses that are incompatible with development of this skill	Below criterion
1	Completely dissatisfied	Staff member displays responses that are incompatible with development of this skill and that are detrimental to client progress	Below criterion

Staff evaluation occurs in an ongoing manner by the trainer using components of the staff evaluation protocol during training sessions. However, a complete evaluation is conducted twice a year to assess staff clinical and professional performance, client performance, and skills in graphing/analyzing data. A practice opportunity (pre-evaluation) is conducted at least 3 months after an instructional staff member is hired in every ASAI agency. This assessment is conducted by the trainer assigned to the classroom. It provides an opportunity for the staff member to experience the process, ask questions, and gain feedback from his/her direct trainer prior to the formal evaluation. It also allows a trainer to obtain data on each staff member’s skill set and to identify and prioritize upcoming training opportunities.

The formal professional staff evaluation is conducted at least 6 months after the instructional staff member begins employment. This assessment is conducted by a trainer who is not directly assigned as the classroom trainer. This promotes a higher level of accountability in the process, such that the outcome from the evaluation can be used to measure both the instructional staff member’s and classroom trainer’s effectiveness. This is part of the quality management process similar to that suggested by Silbaugh and El Fattal in Step 5. In addition, on a number of evaluations two evaluators are assigned to allow for the collection of interobserver agreement data on all measures of the protocol. The data collected across agencies consistently meet or exceed the 80% criterion agreed upon by all agencies. All evaluators use the standardized evaluation protocol, taking observational data and completing all of the protocol sections. For each section, a score is provided based upon direct observational measures and/or the percentage of items within an area rated above, at, or below criterion. The evaluator then summarizes the data as the percentage of evaluation areas above, at, or below criterion to determine if the staff member has passed the evaluation (i.e., a minimum of 80% of the evaluation areas being at criterion). It is important to note that, in each of the agencies, appointment for the upcoming year is based upon passing this evaluation. Only those instructional staff members who pass the evaluation will be invited back as team members the following year—a powerful contingency between performance and employment.

The percentage of staff who pass an evaluation is summarized both within classrooms and across the agency to assess adherence to consistent professional standards. As such, all ASAI agencies annually report the percentage of staff members who were evaluated and, as one QD-KPI, the percentage of those staff members who demonstrated criterion skills in delivering services to clients by passing their evaluation. As seen in Table 6, the mean annual percentage of staff evaluated in each ASAI agency was close to 100% for the 10-year time frame, except for one agency below the 80% criterion. It should be noted that the lowest percentages for all agencies on this QD-KPI occurred during the 2020 year due to the pandemic and virtual instruction replacing in-person instruction for a period (with the full members evaluating 62%, 100%, 65%, 17%, and 0% of their staff, respectively). Despite this disruption, the high mean annual percentage of staff passing their evaluation support the organizations’ commitment to operating with low trainer-to-staff ratios; effectiveness of the training protocol; and staff’s demonstration of requisite skills for delivering effective intervention services and meeting professional standards, including those mentioned by Silbaugh and El Fattal (2022) such as the use of evidenced-based and best practices, implementation of the seven dimensions of behavior analysis, and adherence to the BACB code of ethics.

**Table 6:** Staff evaluation data by agency from 2011 to 2021

Organization	Percentage of staff evaluated		Percentage of staff passing evaluation	
	Mean	Range	Mean	Range
Full Member A	92	62–100	99	95–100
Full Member B	100		100	
Full Member C	93	65–100	99	92–100
Full Member D	84	17–100	99	95–100
Full Member E	74	0–100	100	
Affiliate Member	94	93–94	100	

Although it is not necessarily easy to do, an investment of time and energy in developing skillful and positive staff members using a standardized training and evaluation protocol results in those staff members displaying necessary clinical skills. Analysis of the data collected by that staff member on client progress also demonstrates that meaningful outcomes for clients are achieved as a result. Employing highly skilled trainers who are able to display the skills they are responsible for training, along with successful, positive, and frequent training interactions, is essential to any program. These practices produce a positive workplace culture where the focus is on client progress and high-quality service delivery.

### Client Progress

Every client in each ASAI agency has a defined set of comprehensive annual goals determined to be important by the guardians (as all clients are under the age of 21) and agency team, including but not limited to academic, language and communication, social interaction, recreational, independence, self-help, prevocational, and technology goals. These goals are translated into individualized programs that consist of objective response definitions; measurement procedures; and teaching, generalization, and maintenance conditions. Data on client performance are collected, graphed, and analyzed on an ongoing basis to determine progress towards the annual goals. These data are reviewed regularly by instructional staff (daily review), training staff (weekly review), guardians/families (biweekly to monthly review), and program administrators (quarterly review) to assess acquisition of target goals, define needed modifications to teaching procedures to enhance skill acquisition, and advance programming in an efficient manner. Likewise, as part of each instructional staff member’s evaluation, the entire set of client programs that he/she is responsible for is reviewed using a standardized protocol. This is done to determine if a program is (1) individualized; (2) producing desired behavior change; (3) appropriate (based on current research and ethical practices); (4) has quarterly interobserver agreement data; and (5) contains guardian consent, at a minimum. Table 7 lists both the evaluation dimension and the response definition for each. These data are reviewed on a client-by-client basis to determine the quality of programming delivered to that client by the agency and the staff members involved in the educational process. Additional measures include responsivity to the data, maintenance of behavior, and demonstration of generalized behavior change.

**Table 7:** Evaluation protocol for individualized program review and external program review

Evaluation dimension	Response definition
Individualized	A program is scored as individualized if it contains (1) a written response definition; (2) a written description of the data-collection procedure; (3) a written description of the instruction or treatment procedure; and (4) a graph or data summary. In addition, the response definition, measurement procedure, and intervention procedure must be consistent with the information shown on the data summary
Effective	A program is scored as producing behavior change in a desired direction; no behavior change; behavior change in an undesired direction; or can’t ascertain. The evaluator determines whether there has been behavior change by comparing data since the inception of the program and/or various time periods that he/she selects.
Appropriate	A program is scored as appropriate; may continue; or should be stopped immediately based on a review of the data and the evaluator’s own professional ethics, knowledge of the literature in the field, and concern for clients
Quarterly Interobserver Agreement	The evaluator scores whether percentage interobserver agreement or reliability observers’ scores have been plotted on the data summary at least four times during the last 364 days.
Signed consent	The program is scored as having signed consent if a parent or guardian has signed and dated the program within the last 364 days.

### Support to Parents, Guardians, and Caregivers

As organizations dedicated to producing meaningful and socially significant improvement in client behavior, it is important that ASAI agencies involve guardians and family members in the behavior change process (McClannahan et al., 1982; Rohrer et al., 2021). Each ASAI agency is committed to providing support, mentorship, guidance, and training to guardians to promote high-quality functional outcomes. This regular on-going support promotes the generalization of skills from school to home, the acquisition of environment-specific goals, and the ability of families to complete activities together. Each agency assigns an instructional staff member to each client’s family to provide regular home-programming services for the year.

To evaluate the extent of support in the home and community, each ASAI agency annually evaluates the number of hours of home programming and the presence of individualized programs targeting parent-selected goals in the home setting. Data are collected on the number of sessions and hours of one-on-one training that are delivered to a guardian or other caregiver each year to determine if all families receive regular support and mentorship. Data are also collected on the number of individualized home programs for each client within each agency and the percentage of programs leading to desired behavioral outcomes in the home and community. These home programs are similar to the previously described instructional programs in the education setting, in that they target specific client behavioral responses and are documented in the same technologically sound manner to allow for assessment of outcomes. Collectively, these data are used by each agency to evaluate the extent to which the instructional staff have provided ongoing support to families and guardians through regular visits to the home and direct teaching of new target responses in the home and community environments. Table 8 presents the range and annual mean total hours of home programming across the 10-year time span for each ASAI agency across their entire client population, as well as the mean number of home programming hours per client. The range and mean annual total number of home programs are also presented for the same 10-year time span, as well as the mean annual number of home programs per client.

**Table 8:** Home programming data for each agency from 2011 to 2021

Organization	Home programming hours			Home programs		
	Range	Mean	Mean per client	Range	Mean	Mean per client
Full Member A	948–2,763	1,935	62	187–269	245	8
Full Member B	400–1,222	686	26	83–147	119	5
Full Member C	295–1,051	759	27	75–150	116	4
Full Member D	694–3,289	1,692	56	120–208	167	6
Full Member E	369–698	557	28	112–453	315	16
Affiliate Member	236–520	378	34	32–39	36	3

These data indicate some variability in the number of hours of home programming provided across agencies, as well as across years. However, all clearly exceed the ASAI minimum requirement of 22 one-hr visits to the home per year, per client, and at least one active home program per client. A wide variety of factors influence home programming data, including age of the client (e.g., families with young children often require and receive more hours of home programming), accessibility of the guardian/family (e.g., families with two working parents vs. a stay-at-home parent), afterschool activities (e.g., gymnastics class, swimming), and general client/family need. Each agency is committed to providing individualized support based on these variables. The COVID-19 pandemic greatly affected home programming, because many services were provided virtually in the home setting, explaining increases in the number of hours and home programs for some agencies. Outcome data from the home programs is one QD-KPI used to evaluate an ASAI agency and can be found in the section on Performance Indicators and Outcome Standards.

### Consumer Satisfaction

As noted by Wolf (1978), change in client performance can only be deemed important and meaningful when consumers are satisfied with the process and results. To that end, ASAI agencies annually collect data from a number of consumers, including staff, guardians, and governing board members. A QD-KPI for all of the agencies is the percentage of consumers satisfied with service delivery. Consumer ratings and feedback about aspects of the implemented model and leadership are obtained each year allowing the agency to evaluate the need for strategic changes to meet consumer needs and desires, aligning with Step 4 in Silbaugh and El Fattal’s (2022) call to action . In addition, as new strategies are implemented the consumer data collected can be used to ascertain satisfaction and effectiveness of new initiatives.

The process of consumer evaluation involves sending surveys annually to each of the consumer groups with both general questions (e.g., How satisfied are you with the pleasantness and helpfulness of staff?) and consumer-specific questions (e.g., How satisfied are you with the staff evaluation process? or How satisfied are you with the communication you have with your home programmer?). All consumers receive either a paper survey or a digital survey that asks them to answer the questions found in Table 9 using a 7-point Likert scale similar to that in Table 5. Consumer evaluation surveys are sent in the spring of each year. Each consumer is asked to complete the survey, make comments, and return the survey to the evaluation administrator. Follow-up reminders are sent to all consumers to increase returns rates, the criterion is 80%. Return rates typically exceed this criterion for staff members, guardians, and Board of Trustee members. Responses are confidential and only aggregate data are shared with team members to ensure anonymity of the responder. The data are summarized by agency for both the satisfaction ratings and the written comments from the consumers themselves. The review of agency data allows administrators to determine if the agency is meeting strategic goals and identify future changes in strategy, when necessary, to improve consumer satisfaction. This is a necessary step for organizational leadership to take to ensure analysis of performance and feedback into the system, as suggested by Silbaugh and El Fattal (2022) in Step 6 of their call to action.

**Table 9:** Consumer evaluation survey information

Consumer group	Evaluation area	Question
Guardians	Familiarity	How familiar are you with your child’s instruction, treatment, and home programs?
	Staff cooperation	How satisfied are you with the amount of cooperation and assistance that you receive from Education Program personnel?
	Effective treatment	How satisfied are you that the Education Program personnel are doing an effective job in helping your child (ren)?
	Communication	How satisfied are you with the level of communication you have with Education Program personnel? Do you feel that you can talk freely with them and call them about any problem you may have with your child (ren)?
	Pleasantness	How satisfied are you with the pleasantness of Education Program personnel?
Colleagues	Cooperation	How satisfied are you with amount of cooperation you have received from this person?
	Communication	How satisfied are you with the amount of communication between this person and yourself?
	Pleasantness	How satisfied are you with the pleasantness of the interaction you have with this person?
	Professionalism	How satisfied are you that this person gives and receives feedback in a professional manner?
Program Administration	Use of activity schedules	How satisfied are you with the learners’ planned activities and activity schedules?
	Data collection	How satisfied are you with the number and quality of ongoing data collection activities?
	Opportunities to develop skills	How satisfied are you with your opportunities to develop new skills?
	Training services	How satisfied are you with the training you have received during this past year?
	Evaluation Services	How satisfied are you with the evaluation services you have received during this past year?
	Program responsiveness	How satisfied are you that the program is responsive to your suggestions and input?
	Administration of program	How satisfied are you with the Director’s administration of the program?
Board of Trustees	Cooperation	How satisfied are you with the amount of cooperation you have received from program personnel in their interactions with you?
	Communication	How satisfied are you with the level of communication you have with program personnel?
	Pleasantness	How satisfied are you with the pleasantness of the interactions you have with program personnel?
	Effectiveness	How satisfied are you that the Education Program Staff are providing effective instruction and treatment services?
	Headquarters	How satisfied are you that the building is clean, attractive, safe, and in good repair?
	Staying within guidelines	How satisfied are you that the Directors and Business Manager stay within the guidelines established by the Board of Trustees?
	Leadership	How satisfied are you with the Director’s leadership and program administration?

## Performance Indicators and Outcome Standards

Accountability is necessary in any autism intervention agency. In the absence of performance indicators and outcome standards linked to each member of the organization, it is too easy to avoid responsibility for change. High-quality programs must allocate responsibility and accountability by all staff members to ensure positive client outcomes (McClannahan & Krantz, 1981). It is important to note that ASAI has a “yoked contingency” accountability system in which all staff—from those providing direct service, to the agency directors—assume responsibility (McClannahan & Krantz, 1993).

Each client’s progress must be evaluated to determine his/her attainment of goals. This is completed multiple times each year by the trainer responsible for a particular classroom. This is also completed annually by a different trainer to measure reliability as part of the instructional staff member’s annual evaluation and used to determine criterion performance of that instructional staff member. It is equally as important, however, that the entire agency’s client progress data be evaluated to determine if *the agency* is achieving a benchmark level of performance, a hallmark of a quality program (Autism Commission on Quality, 2022). This QD-KPI provides valuable information about the overall success of the education program in changing the behavior of the client population. To accomplish this, each ASAI agency invites an external evaluator with expertise in behavior analysis and autism intervention to review an evaluator-selected sample of the entire client progress data set at the end of each program year. The evaluator randomly selects and reviews between 10% and 15% of the individualized skill development programs, 35%–45% of the home programs, and 100% of the behavior decrease programs for each client based on the evaluation dimensions presented in Table 7. ASAI’s benchmark for this measure is a minimum of 80% of programs scored as individualized, effective, and appropriate. Interobserver agreement is also collected during the external review, with a minimum of 30% of programs scored by both the external evaluator and trainers from the program to ensure reliability. Interobserver agreement data across all programs is consistently at or above 80% for the dimensions of individualized, effective, and appropriate.

Table 10 presents (1) the mean annual number and range of skill acquisition programs and behavior decrease programs and (2) the mean annual percentage and range of programs evaluated by an external evaluator for the entire client population at each of the agencies across the 10-year period. These data provide important information about not only the external evaluation results, but also the emphasis of programming at the various agencies. Of specific importance is the ratio of skill acquisition programs to behavior decrease programs, which allows an agency to evaluate the extent to which the programming emphasis is on building skill repertoires rather than on decreasing behavior that interferes with learning. Although behavior reduction programs are necessary at times, it is important that an agency evaluate its strength in teaching clients new skills that ultimately result in their participation in a wide range of activities (which is often accompanied by decreases in challenging behavior). The data reflect that each ASAI agency had a substantially larger number of skill acquisition programs than behavior decrease programs.

**Table 10:** Individualized client program data for each agency from 2011–2021

Organization	Number of skill acquisition programs per year		Percentage of skill acquisition programs reviewed per year		Number of behavior decrease programs per year		Percentage of behavior decrease programs reviewed
	Mean	Range	Mean	Range	Mean	Range	Mean
Full Member A	1099	867–1,280	16	8–65	12	4–24	100
Full Member B	645	567–813	22	16–33	12	10–14	100
Full Member C	1029	867–1,189	12	9–17	15	9–23	100
Full Member D	614	534–667	20	13–29	27	17–42	100
Full Member E	912	304–1,262	62	40–100	8	3–11	100
Affiliate Member	258	125–390	65	30–100	7	4–10	100

Another QD-KPI produced from this process is the percentage of evaluated programs that were scored as producing desired behavior change. As evident in Table 11, all ASAI agencies achieved the 80% criterion for the mean percentage of school skill acquisition programs scored as producing desired behavior change. Examples of skill acquisition programs from a variety of skill domains include imitation, conversation, handwriting, mathematical computations, model building, playing soccer, food preparation, self-monitoring, vacuuming, money management, and creating a PowerPoint presentation. As evident in Table 11, there was little to no variability in this QD-KPI among the ASAI agencies.

**Table 11:** Client outcome data for each agency from 2011–2021

Organization	Percentage of Effective Skill Acquisition Programs		Percentage of Effective Behavior Decrease Programs		Percentage of Effective Home Programs	
	Mean	Range	Mean	Range	Mean	Range
Full Member A	93	85–99	71	43–100	91	88–95
Full Member B	98	94–100	95	83–100	96	91–100
Full Member C	94	88–99	90	74–100	86	77–94
Full Member D	98	97–100	86	71–100	96	92–100
Full Member E	99	99–100	93	75–100	99	96–100
Affiliate Member	92	89–94	85	70–100	78	74–81

Table 11 also presents the mean annual percentage of behavior decrease programs scored as producing desired behavior change for each organization across the 10 years. Examples of behavior decrease programs include programs targeting behavioral excesses such as eloping, aggression (e.g., hitting, kicking, throwing objects), and self-injurious behavior (e.g., biting, hitting head, pinching skin).

Five of the six ASAI agencies achieved the benchmark of 80% of the programs being scored as producing desired behavior change when reviewing the means over the 10-year time frame. There is slightly more variability in these data relative to the skill acquisition data, both within and across agencies. Although the scope of this article does not allow for a full discussion of variables influencing that variability, as noted earlier, the number of behavior decrease programs is comparatively small, which may have affected the overall percentage of the programs scored as effective in the event of a single program being scored as not producing desired behavior change. For one agency (full member A), the mean was slightly below criterion, and there was relatively high variability in the annual scores as indicated by the range. In all ASAI agencies, a subcriterion score sets the occasion for review of specific programs and the associated data, collegial discussion about needed changes, and the introduction of potential changes in intervention procedures to increase the percentage of programs scored as producing desired behavior change in the upcoming year.

Finally, the mean percentage of home programs scored as producing desired behavior change across the 10 years can be seen in Table 11. Likewise, five of the six agencies produced mean scores at criterion level, and one of the agencies (affiliate member) reported a slightly subcriterion mean score. The variability in these data among agencies was also quite low.

## Monitor Progress Toward Attainment

Data collected by the ASAI agencies are used to evaluate performance of clients, instructional staff, training staff, leadership, and the organization as a whole (McClannahan & Krantz, 1993, 1997, 2001). In each agency there are a series of yoked contingencies between performance of one member of an agency and performance of another member of the agency. The data collected on client performance are used to monitor progress of instructional staff in acquiring the skills necessary to deliver high-quality services. When clients meet goals, teaching staff are rewarded. When teaching staff meet their goals, trainers are rewarded. And, in turn, when the training staff meet criterion, the leadership team is rewarded. It is important to note that rewards are not material items (such as gift cards or small trinkets) but rather professional opportunities (such as attending a professional conference, presenting at a staff meeting, taking part in a research project). Rewards are individualized in accordance with staff preference and goals to ensure functional and valued rewards. A culture of recognizing accomplishments and rewarding those accomplishments through meaningful professional rewards is a valued part of the accountability system, aligning with Silbaugh and El Fattal’s (2022) suggestion in Step 5, to develop a totally quality management system.

The success of each person within an agency is thus tied to the successful performance of other members of the agency as well as directly to client outcomes. In addition, the success of the organization in coming into contact with positive consequences from its receiving partners results from the collective practices of its staff. Consequences, such as increased client referrals, individual donor contributions, grant and foundation gifts, and invitations to present at national and international conferences, reinforce the team response repertoires that result in positive outcomes for clients. Organizational cultural practices are prompted and shaped to increase access to reinforcing consequences from the stakeholders and the sending educational agencies. Examples of these organizational practices include a well-established system of reciprocal feedback within the organization across all staff members, sharing positive achievements (such as “one thing taught” at regular classroom and staff meetings), attending fundraising events to interface with donors and support the organization, and a culture that emphasizes selection of models based on strong skill repertoires and not simply similar circumstances (e.g., a new instructional staff member will be paired with a senior staff member for an in-service role play, rather than with another new instructional staff member).

This system of yoked contingencies ensures that the delivery of high-quality services lies within the hands of all. As data are collected throughout a given year, the leaders of the organization are able to ascertain progress towards its goals. Data from individual staff evaluations are summarized by classroom to determine success of the trainer in preparing the classroom staff to deliver effective services. Review of the client outcome data by an external evaluator is separated by classroom to evaluate performance of the teaching staff and the trainer. As a whole, the agency data are analyzed to determine effectiveness of the entire training team and the leadership team. Consumer evaluation data are used to ascertain progress of the team in ensuring consumer satisfaction. All of the data collected by the organization, including obtainment of fiscal goals, are used to determine the effectiveness of the executive director. An important contingency exists in that all members of the team—from the teaching staff to the executive director—are appointed annually, with reappointment, salary increases, opportunities to develop new skills, and promotion dependent upon the data obtained from the various sources. In addition, the annual data are collected and shared with other ASAI agencies, as well as individual groups of consumers, aligning with Step 6 of Silbaugh and El Fattal’s (2022) call to action. The public presentation and accountability inherent in this level of data review and data sharing establishes contingencies between performance and professional rewards from consumers, placement agencies, and contributors. It also provides a transparent system open to feedback and honest in its review.

## Professional Performance and Consumer Satisfaction

A requirement for evaluating service delivery is the assessment of organizational performance through consumer feedback. Ensuring that consumers are satisfied with both the process and the outcomes is part of the commitment to ensuring social validity. In Table 12, the mean annual guardian ratings across evaluation areas for each of the agencies for the 10-year period can be seen. A score of 6 (satisfied) is considered criterion. The data demonstrate consistent mean satisfaction ratings from guardians across all agencies for the time period.

**Table 12:** Guardian evaluation data for each organizations from 2011 to 2021

Evaluation area	Organization					
	Full Member A	Full Member B	Full Member C	Full Member D	Full Member E	Affiliate Member
Familiarity	6.7	6.7	6.6	6.7	6.8	6.2
Staff cooperation	6.7	6.8	6.7	6.7	6.8	6.3
Effective treatment	6.7	6.8	6.7	6.7	6.8	6.0
Communication	6.7	6.8	6.8	6.7	6.8	6.7
Pleasantness	6.9	7.0	6.9	6.9	6.9	6.5

Involving staff in the consumer evaluation process is important in any agency. Staff are responsible for implementing strategies, ensuring client progress, and contributing to a positive team and work culture. At the ASAI agencies, each staff member is asked to provide consumer feedback on every other staff member in the agency (including the executive director) through a colleague consumer evaluation survey. Using the 7-point Likert scale, all staff rate their satisfaction with every other colleague’s (1) cooperation; (2) communication; (3) pleasantness; and (4) professionalism, including skills in delivering and receiving feedback (see Table 9 for details). As can be seen in the top panel of Table 13, the mean annual staff ratings for their colleagues across each agency consistently exceeded criterion. Staff are also asked to rate the program and its implementation of various strategies annually. They are asked to rate their satisfaction with the (1) use of activity schedules in the program; (2) data collection activities; (3) opportunities to develop new skills; (4) training services; (5) evaluation services; (6) program responsiveness; and (7) overall administration of the program. In the bottom panel of Table 13, all agencies produced criterion mean annual ratings from staff in the program on this evaluation tool across the 10-year time period.

**Table 13:** Colleague and program evaluation data across agencies from 2011 to 2021

Evaluation area	Full Member A	Full Member B	Full Member C	Full Member D	Full Member E	Affiliate Member
Colleague evaluation						
Cooperation	6.8	6.9	6.8	6.8	6.9	6.7
Communication	6.8	6.9	6.8	6.8	6.9	6.7
Pleasantness	6.8	6.9	6.8	6.8	7.0	6.7
Professionalism	6.8	6.9	6.8	6.8	6.9	6.7
Program evaluation						
Use of activity schedules	6.8	6.8	6.7	6.7		6.8
Data collection activities	6.7	6.8	6.7	6.7		6.6
Opportunities to develop skills	6.5	6.7	6.5	6.5		6.8
Training services	6.5	6.6	6.5	6.5		6.6
Evaluation services	6.8	6.8	6.5	6.5		6.4
Program responsiveness	6.5	6.7	6.5	6.5		6.4
Administration of program	6.7	6.7	6.6	6.6		6.6

Full member E does not complete a program evaluation

A final consumer group valued in many nonprofit organizations (as are several of the ASAI agencies) is the governing board. Three of the ASAI agencies are independent nonprofits and have board of trustees. Governing boards must understand the strategies implemented by the agency leadership and must have an opportunity to provide feedback to the leader of the organization. In addition, consumer evaluation tools prompt attention to important behavior on the part of the program administrator, such as sharing information about client successes, introducing board members to staff members, and providing opportunities for board members to be in the building where services are delivered. The three ASAI agencies with governing boards invite annual feedback from their boards of trustees. Data are collected using the 7-point Likert scale and the governing board is asked to rate their satisfaction with (1) cooperation from the staff; (2) communication with the staff; (3) the pleasantness of the staff; (4) effectiveness of the intervention program; (5) the cleanliness and safety of the headquarters; (6) the director’s adherence to guidelines set by the board; and (7) the overall leadership of the organization. As seen in Table 14, the three agencies with governing boards produced criterion annual mean ratings across all evaluation components across the 10-year time frame.

**Table 14:** Board of trustee evaluation data for each organization from 2011 to 2021

Evaluation area	Organization					
	Full Member A	Full Member B	Full Member C	Full Member D	Full Member E	Affiliate Member
Cooperation	6.8		7.0	6.8		
Communication	6.7		6.9	6.7		
Pleasantness	6.8		7.0	7.0		
Effectiveness	6.6		7.0	6.9		
Headquarters	6.7		6.9	6.9		
Staying within guidelines	6.7		7.0	6.8		
Leadership	6.6		7.0	6.8		

Only Full members A, C, and D have boards of trustees

## Annual Meeting for Evaluating Key Performance Data

A commitment to reviewing and sharing annual performance data holds an organization accountable to its practices, as suggested by Silbaugh and El Fattal (2022) in Step 6 of their call to action. Each ASAI agency uses strategies to review progress towards achievement of standards. Weekly meetings between a trainer and a staff member promote accountability to making required changes in client programming and prompt discussion about family support strategies. Weekly meetings between all members of a classroom results in a permanent product called a “Weekly Individualized Progress Report” that lists tasks and dues dates for each member of the team, another accountability strategy. Weekly meetings between members of the management team take place, using the same tool to increase consistent training efforts across the program and advancement towards organizational goals. Each of the above-mentioned meetings results in a permanent product that is circulated to all involved members and the leadership of the organization.

Staff evaluation results in a written product used by the instructional staff member and trainer to set training goals for that instructional staff member. Likewise, the external review of the data notebooks within the agency results in a written product that is used by the team to make changes to client programming and agency policy and procedures. Items identified from these summaries are incorporated into the Weekly Individualized Progress Reports noted above to prompt both action and follow-up.

Each ASAI agency prepares an annual report that contains data on all measures described herein, as well as many others. ASAI holds an annual meeting at which each agency shares their data set, notes accomplishments (relative to the prior year’s data and goal setting), and identifies areas in need of improvement and/or new strategies that are being introduced as result of the outcome data. Collective conversations and feedback from other member agencies promote a team-approach and advancement of the model that we all operate from. High-quality service delivery relies upon system improvement as a result of connections between data collected and the systems implemented. The annual collection, presentation, and discussion of each agency’s data promotes another level of accountability in the system. Each agency must meet criterion on the data measures collected and/or show a positive trend towards criterion in those areas where progress was needed to maintain its membership in ASAI.

The data are also shared by each agency with specific consumer groups, such as governing board members, guardians/families, and staff. Public data sharing promotes transparency and accountability to consumers, as well as provides opportunities for stakeholder input and discussion among colleagues to promote change. This is in accordance with Step 6 in Silbaugh and El Fattal’s (2022) call to action.

## Summary

Silbaugh and El Fattal (2022) provided an accurate and needed call to action. As they noted, autism service delivery providers are obligated to use the science of ABA to define intervention quality and build systems to ensure the implementation of key variables and strategies consistently and that sustain over time. It is ASAI’s position that these variables must promote both professional competence and consumer satisfaction, which are directly linked to service delivery.

This article presents data from six independent agencies, all of whom implement the same model of intervention based on the work of McClannahan and Krantz (1993). The data presented support both the fidelity of implementation of the model, as well as the production of competency in professionals that translated into positive and meaningful client outcomes. Data on ASAI agency performance in training instructional staff to criterion and building new response repertoires in clients were consistent across the five full member agencies, year after year, for the 10 years assessed. Data from a developing program (i.e., the ASAI affiliate member) demonstrated remarkable similarity, even after only 2 years of operation. Taken together, these data support the robustness of the ASAI model in establishing QD-KPIs, contingencies within the organization to promote attainment of performance standards, regular data-based methods of monitoring performance on an ongoing basis and across years, and advancement of the system components based on the data collected.

The six-step call to action outlined by Silbaugh and El Fattal (2022) prompts ABA service delivery providers to base their organizational practices on systems that promote consistency in implementation and reinforcement of organizational behavior that produces positive client outcomes while ensuring the financial stability of an organization. ASAI member agencies begin with strategic planning (Step 1 defined by Drs. Silbaugh and El Fattal), identifying QD-KPIs based on professional performance, positive client outcomes, and consumer satisfaction. They also implement defined organizational systems and strategies to attain stated goals, while ensuring fiscal responsibility that includes balancing budgets and planning achievable fundraising goals to ensure quality of services. As all of the agencies are education programs, funding is acquired through local districts and fundraising efforts.

Increasing enrollment at agencies increases tuition income, but also places a financial demand on the organization as well. Delivering high-quality services with the program components identified in this article (e.g., low trainer to instructional staff ratio, low instructional staff to student ratio, frequent hands-on training and evaluation by dedicated training staff, regular data analysis and program modification) increases the cost per student. Thus, strategic plans at the ASAI agencies must include determinations about increasing enrollment or additional services (e.g., provision of adult services or early intervention) while maintaining criterion levels of performance that align with the ASAI Standards of Excellence and fundraising achievements that provide the needed resources to support positive outcomes.

The QD-KPIs are tied to both professional standards that are promoted and measured through a structure performance evaluation protocol based on best practice and ethical service implementation and consumer satisfaction with both outcomes and service delivery (Step 2). The annual collection of data in accordance with the ASAI Standards of Excellence across years allows the program administrators to monitor and assess progress towards the fulfillment of the strategic plan (Step 3). The professional evaluation protocol originally developed by McClannahan and Krantz (1993) is continually updated based upon current professional practice, with new sections added to the protocol to allow for both teaching and evaluation of core competencies identified by professional organizations, such as the BACB and BHCOE, as well as advances in our field. Likewise, consumer surveys align with defined standards of what is valued by consumer groups. These tools provide the organizations with a method of both collecting data and analyzing attainment of QD-KPIs (Step 4). The ASAI agencies engage in regular practices to promote quality, including promoting effective communication via a standardized multidirectional structured system that all staff are expected to use, developing a culture of positive practices to promote problem solving and an emphasis on achieving excellence, and contingently rewarding those professional repertoires that align with success in achieving positive goals for clients, consumers, and the organization (Step 5). Lastly, there is a system of accountability in each organization whereby the data that are collected are regularly shared with and evaluated by not only the agency, but other members of ASAI and the consumers of the organization’s services (Step 6).

Although ASAI promotes excellence in autism intervention, promoting excellence alone is not sufficient. The members of ASAI have developed, implemented, and refined program components to produce positive outcomes and consumer satisfaction by various groups, along with contingencies for supporting the sustained performance of the organization. This is exactly what Silbaugh and El Fattal (2022) encouraged autism intervention providers to do. McClannahan and Krantz encouraged the same in their 1993 article on systems analysis. The data presented in this article are a mere sample of the numerous outcomes measures which ASAI members have collected and presented to one another for decades. We anticipate publishing additional data on the generality of these systems to other autism intervention organizations (e.g., public schools, for-profit in-home providers) in the future.

Variability in service delivery is unfortunately common in the autism service delivery field, as suggested by Silbaugh and El Fattal (2022). Program administrators struggle with reducing variability in staff practices, organizational culture, and client outcomes. We have learned from our multi-agency initiative that the development of a standardized set of expectations, training tools, evaluation tools for both professional staff performance and client outcomes, and communication among team members can be used to develop a more consistent set of practices and positive organizational culture that values and reinforces meaningful client outcomes. This aligns with other statements from professional organizations, such as CASP and BHCOE. As an organizational leader, ASAI emphasizes that systems like those described in this article must be developed and implemented consistently. Performance data from individual clients, professional staff members, the aggregate client body, and the organization itself can be reviewed and used to ascertain effectiveness in key areas and to identify next steps for the organization along with a strategic plan to accomplish change. In addition, contingencies within the organization must be arranged to promote both attention to and achievement of QD-KPIs (especially those of positive client outcomes and consumer satisfaction).

In the absence of strong contingencies of reinforcement between staff behavior, organizational practices, client outcomes, and consumer satisfaction, it is likely that other contingencies will shape the future of our field. Behavior analysts are in high demand and intervention programs are in even higher demand. It is time for us to use our science to inform our practices and shape responsible and accountable agencies that deliver consistent intervention that produces meaningful client behavior change and satisfied consumers, rather than solely generate income.

Additional study is needed relative to the particulars of the many program components briefly touched on in this article, along with data that support assertions of excellence of one method over another. It is also important that dissemination efforts are better defined and studied to allow for improved assessment of key program components, components that provide some value but may not be critical to outcomes, cultural differences and how they affect the development of models, and the process of shaping new organizations in the service delivery realm. ASAI looks forward to contributing to this area in the future along with our colleagues to accomplish our mission of preserving, disseminating, and enhancing services for autistic individuals.

## Data Availability

The data sets that were analyzed and summarized for the current article are available from the corresponding author on reasonable request.

## Declarations

### Publication Submission

This article has not been previously published and has not been or will be submitted elsewhere during the review process.

## References

[CR1] Autism Commission on Quality. (2022). Applied behavior analysis accreditation program standards and guides. https://autismcommission.org/standards. Accessed 29 June 2023

[CR2] Baer DM, Wolf MM, Risley TR (1987). Some still-current dimensions of applied behavior analysis. Journal of Applied Behavior Analysis.

[CR3] Behavioral Health Center of Excellence. (2021). Selecting appropriate assessment instruments to measure treatment outcomes for individual with autism spectrum disorder: Guidelines for practitioners, payors, patients, and other stakeholders. *Author.*

[CR4] Cohen H, Amerine-Dickens M, Smith T (2006). Early intensive behavioral treatment: Replication of the UCLA model in a community setting. Developmental & Behavioral Pediatrics.

[CR5] Council of Autism Service Providers. (2020). Applied behavior analysis treatment of autism spectrum disorder: Practice guidelines for healthcare funders and managers. https://assets-002.noviams.com/novi-file-uploads/casp/pdfs-and-documents/ASD_Guidelines/ABA-ASD-Practice-Guidelines.pdf. Accessed 29 June 2023

[CR6] Dixon MR (2014). The next generation of ABA providers. Behavior Analysis in Practice.

[CR7] Eldevik S, Eikeseth S, Jahr E, Smith T (2006). Effects of low-intensity behavioral treatment for children with autism and mental retardation. Journal of Autism & Developmental Disorders.

[CR8] Ellis J, Glenn SS (1995). Behavior-analytic repertoires: Where will they come from and how can they be maintained?. The Behavior Analyst.

[CR9] Fenske E, Zalenski S, Krantz PJ, McClannahan LE (1985). Age at intervention and treatment outcome for autistic children in a comprehensive intervention program. Analysis and Intervention in Developmental Disabilities.

[CR10] Glenn S, Malott M (2004). Complexity and selection: Implications for organizational change. Behavior & Social Issues.

[CR11] Handleman JS, Harris SL (2005). Douglass developmental disabilities Center: An ABA program for children and adults with autism spectrum disorder. International Journal of Behavioral & Consultation Therapy.

[CR12] Howard JS, Sparkman CR, Cohen HG, Green G, Stanislaw H (2005). A comparison of intensive behavior analytic and eclectic treatments for young children with autism. Research in Developmental Disabilities.

[CR13] Hume K, Steinbrenner JR, Odom SL, Morin KL, Nowell SW, Tomaszewski B, Szendrey S, McIntyre NS, Yucesoy-Ozkan S, Savage MN (2021). Evidence-based practices for children, youth, and young adults with autism: Third generation review. Journal of Autism & Developmental Disorders.

[CR14] Krantz, P. J., & McClannahan, L. E. (1999). Behavior analysis in autism intervention. Address at Society for the Advancement of Behavior Analysis (SABA) Awards ceremony at the meeting of the Association for Behavior Analysis, Chicago, IL.

[CR15] McClannahan, L. E., & Krantz, P. J. (1981). Accountability systems for protection of the rights of autistic children and youth. In G. T. Hannah, W. P. Christian, & H. B. Clark (Eds.), Preservation of client rights (pp. 84–106). Free Press.

[CR16] McClannahan LE, Krantz PJ (1993). On systems analysis in autism intervention programs. Journal of Applied Behavior Analysis.

[CR17] McClannahan LE, Krantz PJ (1997). Princeton child development institute. Behavior & Social Issues.

[CR18] McClannahan, L. E., & Krantz, P. J. (2001). Behavior analysis and intervention for preschoolers at the Princeton child development institute. *Preschool education programs for children with autism*, 191–213.

[CR19] McClannahan, L. E., & Krantz, P. J. (2005). *Disseminating autism intervention technology: Don’t go froth and mortify [invited address]*. CA: CalABA Annual Convention.

[CR20] McClannahan LE, Krantz PJ, McGee GG (1982). Parents as therapists for autistic children: A model for effective parent training. Analysis & Intervention in Developmental Disabilities.

[CR21] McGee GG, Morrier MJ, Ala'i-Rosales S (2020). Contributions of university lab schools to behavior analysis. European Journal of Behavior Analysis.

[CR22] Odom SL, Hall LJ, Suhrheinrich J (2020). Implementation science, behavior analysis and supporting evidence-based practices for individuals with autism. European Journal of Behavior Analysis.

[CR23] Parsons MB, Rollyson JH, Reid DH (2012). Evidence-based staff training: A guide for practitioners. Behavior Analysis in Practice.

[CR24] Reid DH, Parsons MB, Jensen JM (2017). Maintaining staff performance following a training intervention: Suggestions from a 30-year case example. Behavior Analysis in Practice.

[CR25] Rohrer JL, Marshall KB, Suzio C, Weiss MJ (2021). Soft skills: The case for compassionate approaches or how behavior analysis keeps finding its heart. Behavior Analysis in Practice.

[CR26] Sherman J, Richardson J, Vedora J (2021). The use of behavioral skills training to teach components of direct instruction. Behavior Analysis in Practice.

[CR27] Silbaugh BC, El Fattal R (2022). Exploring quality in the applied behavior analysis service delivery industry. Behavior Analysis in Practice.

[CR28] Slocum TA, Detrich R, Wilczynski M, Spencer TD, Lewis T, Wolfe K (2014). The evidence-based practice of applied behavior analysis. The Behavior Analyst.

[CR29] Smith T, Groen AD, Wynn JW (2000). Randomized trial of intensive early intervention for children with pervasive developmental disorder. American Journal on Mental Retardation.

[CR30] Williams, B. F., & Williams, R. L. (2010). Princeton child development institute: Across the lifespan. In B. F. Williams & R. L. Williams (Eds.), *Effective programs for autistic spectrum disorder: Applied behavior analysis models* (pp. 195–209). Routledge. 10.4324/9780203855034-12

[CR31] Wolf MM (1978). Social validity: The case for subjective measurement of how applied behavior analysis is finding its heart. Journal of Applied Behavior Analysis.

